# Identification of *Malassezia furfur* Secreted Aspartyl Protease 1 (MfSAP1) and Its Role in Extracellular Matrix Degradation

**DOI:** 10.3389/fcimb.2020.00148

**Published:** 2020-04-09

**Authors:** Si En Poh, Joleen P. Z. Goh, Chen Fan, Wisely Chua, Shi Qi Gan, Priscilla Lay Keng Lim, Bhavya Sharma, David I. Leavesley, Thomas L. Dawson, Hao Li

**Affiliations:** ^1^Molecular Engineering Lab, Institute of Bioengineering and Nanotechnology, Agency for Science Technology and Research, Singapore, Singapore; ^2^Skin Research Institute of Singapore, Agency for Science Technology and Research, Singapore, Singapore; ^3^School of Pharmacy, Department of Drug Discovery, Medical University of South Carolina, Charleston, SC, United States

**Keywords:** skin microbiome, *Malassezia*, protease, extracellular matrix, wound healing

## Abstract

*Malassezia* is the most abundant eukaryotic microbial genus on human skin. Similar to many human-residing fungi, *Malassezia* has high metabolic potential and secretes a plethora of hydrolytic enzymes that can potentially modify and structure the external skin environment. Here we show that the dominant secreted *Malassezia* protease isolated from cultured *Malassezia furfur* is an aspartyl protease that is secreted and active at all phases of culture growth. We observed that this protease, herein named as MfSAP1 (*M. furfur* secreted aspartyl protease 1) has a broader substrate cleavage profile and higher catalytic efficiency than the previously reported protease homolog in *Malassezia globosa*. We demonstrate that MfSAP1 is capable of degrading a wide range of human skin associated extracellular matrix (ECM) proteins and ECM isolated directly from keratinocytes and fibroblasts. Using a 3-D wound model with primary keratinocytes grown on human de-epidermized dermis, we show that MfSAP1 protease can potentially interfere with wound re-epithelization in an acute wound model. Taken together, our work demonstrates that *Malassezia* proteases have host-associated substrates and play important roles in cutaneous wound healing.

## Introduction

The skin is our first physical barrier against the external environment and is also the residence of a rich community of microbes (Oh et al., 2014; Byrd et al., 2018). This surface is mainly colonized by diverse groups of bacteria, though intriguingly, fungal members of the skin microbiota are dominated by a single family-*Malassezia* (Findley et al., 2013; Grice and Dawson, 2017). While *Malassezia* is less abundant than skin bacteria, it has much larger biomass that allows functional significance (Ramasamy et al., 2019). This basidiomycete which mainly exists in the yeast form is highly prevalent in sebaceous areas such as scalp, back and facial skin (Prohic et al., 2016; Jo et al., 2017). Advances in sequencing technology have enabled detailed characterization of the genome sequences of skin-residing microbes isolated through both culture-dependent and culture-independent methods (Grice, 2015; Byrd et al., 2017). Functional annotations of the *Malassezia* genome have revealed the presence of many genes encoding for hydrolytic enzymes- namely proteases, esterases (including lipases and phospholipases) and glucosyl hydrolases (Xu et al., 2007; Gioti et al., 2013; Park et al., 2017; Zhu et al., 2017). This is especially relevant for the skin environment which is nutrient-poor and enriched with lipids and proteins (Chen et al., 2018). In particular, proteases are nature's powerful tools in mediating catabolism of proteins (López-Otín and Bond, 2008), where degradation of specific protein targets can function in important processes such as nutrient acquisition and skin surface adherence (Naglik et al., 2003; Wessler et al., 2017).

In our previous work, we determined that the major secreted protease in the skin commensal *Malassezia globosa* is the aspartyl protease MgSAP1 (Li et al., 2018). This protease is readily secreted in microbial culture during exponential growth of *M. globosa* and is able to reduce *Staphylococcus aureus* biofilm formation, partially through cleavage of the *S. aureus* protein A. Genome analysis of other well-characterized *Malassezia* species such as *Malassezia furfur, Malassezia restricta* and *Malassezia sympodialis* further reveals that secreted proteases are well-conserved across the phylum (Wu et al., 2015). This suggests that these secretory enzymes are important for *Malassezia*'s metabolic functions. In the related human fungal opportunistic pathogen *Candida albicans*, production of secreted aspartyl proteases (SAPs) is closely associated with virulence and pathogenesis (Naglik et al., 2003). These secreted *C. albicans* SAPs are capable of degrading many human proteins and this facilitates invasion and colonization of this microbe on mucosal surfaces (Naglik et al., 2008; Winter et al., 2016).

In this study, we focused on characterizing the dominant protease secreted by *M. furfur*. *M. furfur* colonization on skin surfaces is much less abundant than *M. globosa* and *M. restricta*, but it has been associated with various dermatological conditions such as pityriasis versicolor (Gaitanis et al., 2012; Velegraki et al., 2015). More significantly, *M. furfur* is involved in certain rare systemic infections in immunosuppressed patients and in neonates on parenteral nutrition (Gupta et al., 2014; Chen et al., 2019). Functional annotation of the recently sequenced *M. furfur* CBS 14141 genome enabled us to identify different classes of secretory proteases. Using quenched fluorogenic substrates, we determined that the major secreted protease activity in the extracellular media of *M. furfur* is attributed to an aspartyl protease that is a close homolog of the previously characterized MgSAP1 protease in *M. globosa*. This protease, which we herein name *M. furfur* Secreted Aspartyl Protease 1 (MfSAP1), is highly catalytically efficient and processes a broader range of fluorogenic substrates as compared to MgSAP1. We determined that MfSAP1 rapidly cleaves a wide range of extracellular matrix (ECM) proteins associated with the dermis and epidermis. Using an acute wound model created on a 3-D human skin equivalent grown on de-cellularized human dermis, we demonstrated that a high concentration of MfSAP1 can interfere with re-epithelization after wounding.

## Materials and Methods

### Annotation of the *M. furfur* CBS 14141 Secreted Proteases and Dendrogram Construction

*Malassezia furfur* CBS 14141 was sequenced and the genome assembled in our previous study (Wu et al., 2015) (BioProject: PRJNA286710). Putative transcripts and protein sequences were assigned using FUNAnnotate (unpublished data). Protease prediction and assignment of protease families were performed using MEROPS (Rawlings et al., 2018) (https://www.ebi.ac.uk/merops/). Secreted *M. furfur* proteases were predicted using SignalP 5.0 (Armenteros et al., 2019). The previously published list of secreted *M. globosa* CBS 7966 proteases were re-analyzed with the recently updated SignalP 5.0 to generate a revised list of secreted proteases. For dendrogram construction, protein sequences were aligned using Clustal Omega. Maximum likelihood analysis was performed with IQTree (Trifinopoulos et al., 2016) using the default settings with 1,000 bootstraps. The phylogenetic tree was constructed using Dendroscope (dendroscope.org).

### *M. furfur* Culture and Enrichment of Aspartyl Protease

*M. furfur* CBS 14141 (previously named JLPK23) strain was cultured routinely in modified Dixon (mDixon) liquid (shaking at 150 rpm) or agar media at 32°C as reported previously (DeAngelis et al., 2007). Sabourad's Dextrose broth (Sigma Aldrich) was used at 30 g/l with 1% Tween-40 (Sigma Aldrich) supplementation. Minimal media culture was prepared using 3.4 g/l of yeast nitrogen broth without amino acids and ammonium sulfate (BD Difco), 5 g/l ammonium sulfate (Sigma Aldrich), 0.2% glycerol (Sigma Aldrich) and 1% Tween-40 at a final pH of 6. Culture extracellular media was obtained by spinning down the yeast culture at 5000 rpm and filtering the supernatant through 0.22 μm vacuum filter. Aspartyl proteases were enriched from the culture supernatant using pepstatin A-agarose resin (Sigma Aldrich) as previously described (Li et al., 2018). Briefly, prewashed pepstatin A-agarose beads were incubated with the culture extracellular media obtained after the specified time of growth, with shaking at 4°C for 1 h. The beads were subsequently washed with 50 mM sodium citrate and 500 mM sodium chloride at pH 4.2, further washed in 50 mM Tris-HCl pH 5.0 buffer and eluted in 100 mM sodium bicarbonate, 500 mM sodium chloride, pH 10.0. Protein concentration was determined using the Qubit Protein Assay kit (ThermoFisher Scientific).

### Protease Assay With Internally Quenched Fluorogenic Peptides

Screening of protease activity with substrates S1-S19 was performed by diluting the *M. furfur* extracellular media in a final concentration of 2% (by volume) in 50 mM sodium citrate buffer, pH 4.2. The commercially available substrates S1-S19 (CPC Scientific) were used at a final concentration of 20 μM, unless otherwise indicated. The substrate sequences are listed in Supplementary Material. Fluorescence was monitored at Ex/Em = 330/390 nm on a SpectraMax M2 microplate reader (Molecular Devices) and the slope over the linear range of the signal was used to calculate the proteolytic activity. For inhibition assays, each inhibitor was added at the indicated concentration and activity was determined as above, and calculated as a percentage of the control. For K_m_ and k_cat_ determination, enriched MfSAP1 was used at a final concentration of 5 pg/μl with varying S12 substrate concentrations and the parameters were calculated using the Michaelis Menten non-linear regression on Graphpad Prism 8.3.0.

### *In vitro* Extracellular Matrix (ECM) Protein Degradation Assays

ECM proteins were purchased from commercial sources as follows—fibronectin (Sigma Aldrich #F0895), rat tail collagen type I (BD Bioscience, #354236), collagen IV from human placenta (Sigma Aldrich #C5533), keratin from human epidermis (Sigma Aldrich #K0253), laminin from human fibroblasts (Sigma Aldrich #L4544), vitronectin from human plasma (Sigma Aldrich #5051), and human thrombospondin-1 (Sigma Aldrich #ECM002). Degradation of the ECM proteins was assessed by incubating each protein at varying substrate to enzyme ratio of enriched MfSAP1 for 4 h at 34°C. 2X Laemmli sample buffer (Biorad) was added at the endpoint, boiled for 5 min and loaded onto NuPAGE Novex 4–12% Bis-Tris Protein gel (ThermoFisher Scientific) or 4–20% Mini-PROTEAN TGX precast gel (Biorad). The gel was stained with SimplyBlue Safestain (ThermoFisher Scientific).

### Protease Treatment of Decellularized Keratinocyte and Fibroblast Cell Cultures

N/TERT-1 keratinocytes (Dickson et al., 2000) were grown to ~60% confluency in keratinocyte serum free media (Life Technologies) on 10 cm tissue cultured treated dishes and decellularized using freshly made 20 mM ammonium hydroxide following the protocol of Hellewell et al. (2017). Primary dermal fibroblasts were cultured using the previously reported macromolecular crowding model (Lareu et al., 2007) in DMEM (Nacalai Tesque) medium containing 18.78 mg/ml Ficoll 70 (Sigma Aldrich), 12.5 mg/ml Ficoll 400 (Sigma Aldrich), and 100 μM ascorbic acid (Sigma Aldrich). Fibroblasts were decellularized using previously published protocol (Demidova-Rice et al., 2011). Briefly, cell culture media was discarded and the cells were washed with phosphate buffered saline (PBS). Decellularization buffer containing 20 mM Tris pH8 (1st Base), 15 mM sodium chloride, 1 mM ethylene glycol-bis(β-aminoethyl ether)-N,N,N′,N′-tetraacetic acid (EGTA) (Sigma Aldrich), 1 mM phenylmethylsulfonyl fluoride (PMSF) (Sigma Aldrich), and 0.5% w/v sodium deoxycholate (ThermoFisher Scientific) was added for 5 min at room temperature. The remaining ECM was then washed 5 times with PBS. For protease treatment, enriched MfSAP1 in 50 mM sodium citrate buffer pH 4.2 was added at the indicated concentrations and incubated at 30°C for 2 hrs. The remaining ECM was washed 3 times with PBS, hot 2X Laemmli buffer added and the plate was scraped using a cell scraper. The samples were then loaded onto NuPAGE Novex 4–12% Bis-Tris Protein gel and stained using SimplyBlue Safestain.

### Preparation of 3-D Primary Keratinocyte Cultures on De-Epidermized Dermis (DED)

Primary human keratinocytes were obtained from Asian Skin Bank, A^*^STAR, with ethical approval IRB: B-16-135E. The cells were cultured in Full Green's media as previously described with modification (Xie et al., 2010). The cells were maintained at 37°C in an incubator with 5% CO_2_/95% air, with a change of medium every 2–3 days.

The DED-HSE wound healing model was performed as previously published (Xie et al., 2010). Briefly, full thickness skin tissue samples (surgical discard) were purchased from Genoskin, France with ethical approval IRB: B-16-135E. They were trimmed into 1 cm^2^ pieces and submerged in 1 M NaCl overnight. After decellularization, the epidermal layer was removed and sterile stainless-steel rings were placed onto the papillary side of each de-epidermized dermis (DED). Keratinocytes (20,000 cells) were transferred into the center of each ring placed on the DEDs and incubated for 2 days. After 2 days of incubation, the rings were removed and the reconstructed samples were elevated to the air-liquid interface and cultivated for another 9 days. To create a wound, a 4 mm biopsy punch (Integra Miltex) was used to remove the reformed epidermis layer on the samples. Topical treatments of pH 5 Full Green's media, 1 μg/ml MfSAP1, 10 μg/ml MfSAP1 (both prepared in pH 5 Full Green's media) were applied thereafter to the wound area daily for 4 days, from the day of wounding. Wound closure was visualized by performing 3-(4,5-dimethylthiazol-2-yl)-2,5-diphenyltetrazolium bromide (MTT) assay (0.5 mg/ml; Sigma Aldrich) as previously described (Xie et al., 2010), and the images were captured using Nikon SMZ745T microscope. A total of 3 independent experiments were performed. Quantification of the uncovered wound area and lateral migration was performed using ImageJ 1.52a. For each experiment, 2 technical replicates with the nearest values of each sample were used for quantification analysis. Statistical analysis was performed using the two-tailed Mann-Whitney Test on Graphpad Prism 8.3.0. The DED-HSE samples were then fixed and embedded in paraffin for further histological processing and analysis. Details on histology and immunohistochemistry is found in Supplementary Material.

## Results

### Prediction of *Malassezia furfur* Secreted Proteases

**Figure 1:**
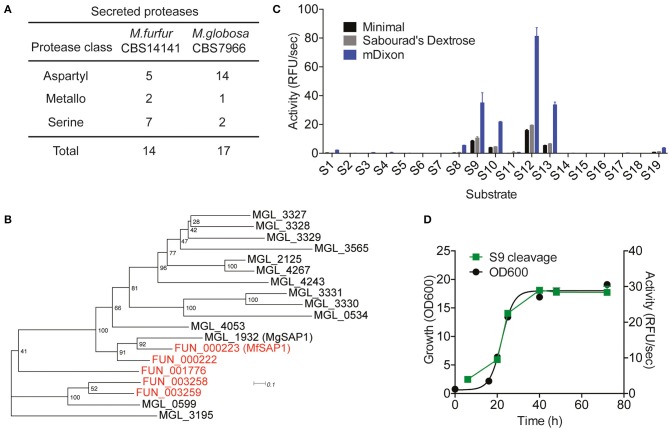
Prediction of *Malassezia furfur* secreted proteases and assessment of extracellular protease activity in culture. **(A)** A comparison of the predicted secreted proteases in *M. furfur* CBS 14141 and *M. globosa* CBS 7966. **(B)** Unrooted phylogenetic tree of the predicted secretory aspartyl proteases in *M. globosa* (14, in black) and *M. furfur* (5, in red). Number at each node indicates the bootstrap value. **(C)** Extracellular protease activity of *M. furfur* cultured in 3 different media assessed using 19 internally quenched substrates. **(D)** Correlation of secreted protease activity using a representative substrate S9 with planktonic culture growth. Error bars represent standard deviation for *n* = 3.

Using the previously sequenced and assembled haploid *M. furfur* CBS 14141 genome, we identified 4132 putative protein-coding genes. These genes were further analyzed using the MEROPS pipeline for protease family assignment and the secreted proteases were predicted using SignalP 5.0. From this, we identified a total of 14 secreted proteases (Figure 1A, Supplementary Table 1). While the overall number of predicted secretory proteases is similar to *M. globosa* CBS 7966, one distinct difference is the expansion of the secreted serine protease family and the reduction of the aspartyl secreted protease family in *M. furfur*.

To compare the 5 *M. furfur* predicted secreted aspartyl proteases with those of *M. globosa*, we performed a multiple sequence alignment using Clustal Omega followed by an unrooted phylogenic tree analysis (Figure 1B). FUN_000223 clusters most closely with the previously characterized MgSAP1 (67.7% similarity), and will herein be named as *M. furfur* Secreted Aspartyl Protease 1 (MfSAP1). FUN_000222 is closely related to both MfSAP1 and MgSAP1, while FUN_003258 and FUN_003259 are more similar to MGL_0599 (Figure 1B).

### Protease Activity in Culture Is Dominated by MfSAP1

To determine secreted protease activity, we cultured *M. furfur* CBS 14141 in 3 different media of varying nutrient richness: mDixon, Sabourad's Dextrose and minimal media. We isolated the extracellular media and performed protease activity assays using a diverse panel of 19 internally quenched fluorescent substrates (S1-S19, see Supplementary Material). We observed that when grown in mDixon, *M. furfur* produced the highest extracellular protease activity and this corresponds to the higher growth density obtained in this rich media (Figure 1C). We detected a similar pattern of substrate cleavage preference in all 3 media, with substrate S12 having the most preferred cleavage sequence. We further assessed the proteolytic activity throughout the planktonic growth phases of *M. furfur*, and observed that the protease activity correlates with growth (Figure 1D). This is in clear contrast with the secreted protease activity from *M. globosa* detected using the same fluorogenic substrate, which peaks at late log phase and decreases thereafter (Li et al., 2018).

**Figure 2:**
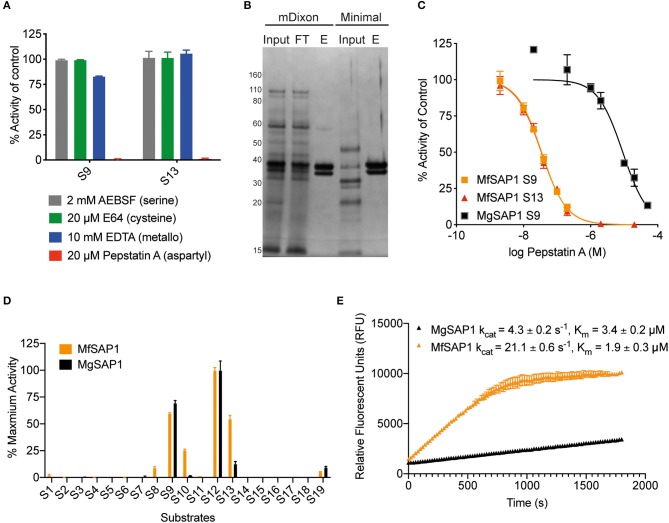
Identification of the major aspartyl protease MfSAP1 in *M. furfur* and comparison with its *M. globosa* homolog MgSAP1. **(A)** *M. furfur* extracellular media was treated with each protease inhibitor and the remaining protease activity was assessed using S9 and S13. **(B)** Silver stain of the extracellular media (input), the enriched elute (E) and the flow-through (FT) from the pepstatin A-agarose affinity purification of the secreted proteases in *M. furfur* culture grown in mDixon and minimal media. **(C)** Inhibition curves of the enriched proteases MfSAP1 and MgSAP1 against the aspartyl protease inhibitor pepstatin A. **(D)** Comparison of the substrate cleavage preferences of MfSAP1 and MgSAP1 for the quenched substrates S1-S19. Protease activities were normalized to the maximum activity of the panel (S12) in each enzyme. **(E)** Kinetic parameters of enriched MfSAP1 and MgSAP1 for cleavage of substrate S12. A representative plot at 20 μM of S12 is shown for both enzymes. The Michaelis-Menten plot for MfSAP1 is included in Supplementary Figure 1. The data for MgSAP1 is previously published in Li et al. (2018). Error bars represent standard deviation for *n* = 3.

To identify the class of protease responsible for the detected activity, we assessed the protease activity of the extracellular media after treatment with class specific protease inhibitors. Inhibitors of serine proteases (AEBSF), cysteine proteases (E-64), and metalloproteases (EDTA) had no significant effect on the protease activities determined using S9 and S13. In contrast, virtually no protease activity was detected in the presence of aspartyl protease inhibitor pepstatin A (Figure 2A). As this indicates that the major secreted enzymes activities are contributed by aspartyl proteases, we used pepstatin A-agarose as a chemical affinity tool to enrich for aspartyl proteases present in the *M. furfur* mDixon and minimal conditioned media. The major protein species we isolated are two proteins around 30–40 kDa (Figure 2B). Using in-gel trypsinization followed by mass spectrometry, we determined that both proteins have the same peptide sequences that corresponds to MfSAP1 (Supplementary Figure 1). We further confirmed that the two proteins have the same N-terminal sequence by Edman sequencing (Supplementary Figure 1). We interpret that the two protein species are likely different glycosylated forms of MfSAP1. We next determined the optimum pH for MfSAP1 and found it to be between pH 4 and 5 (Supplementary Figure 1), commensurate with the acidic pH of healthy skin. From the pepstatin A-agarose enrichment, we estimate that >95% of the protease activity in the culture conditioned media is attributed to MfSAP1, and using purified enzyme activity as an estimation, MfSAP1 is secreted at a concentration of 4.4 μg/ml in culture after late log phase in mDixon media.

### Comparison of *M. furfur* MfSAP1 and *M. globosa* MgSAP1

To compare the dominant secreted protease of *M. furfur* and *M. globosa*, we first determined the IC_50_ of pepstatin A inhibition from enriched preparations of both enzymes. To our surprise, the IC_50_ for MfSAP1 as determined by inhibition of substrate S9 is 35 ± 1 nM, which is much lower than the IC_50_ of 9 ± 1 μM for MgSAP1 (Figure 2C). The stronger preference of pepstatin A in inhibiting MfSAP1 over MgSAP1 suggests that there may be substantial differences in the active site binding pocket of these enzymes. To verify this, we assessed the substrate cleavage preference of both enzymes using the panel of 19 substrates. We observed that while the overall trend of preferred substrates is similar for MfSAP1 and MgSAP1, MfSAP1 cleaves an expanded repertoire of these fluorogenic substrates (Figure 2D). This is evident in MfSAP1's enhanced cleavage of S8, S10 and S13 compared to MgSAP1. We next determined the catalytic efficiency of both enzymes using the most preferred substrate S12 and observed that the k_cat_/K_m_ for this substrate is nearly 10-fold greater for MfSAP1 than that for MgSAP1 (Figure 2E, Supplementary Figure 1).

### MfSAP1 Readily Cleaves Human Skin-Associated Extracellular Matrix Proteins

**Figure 3:**
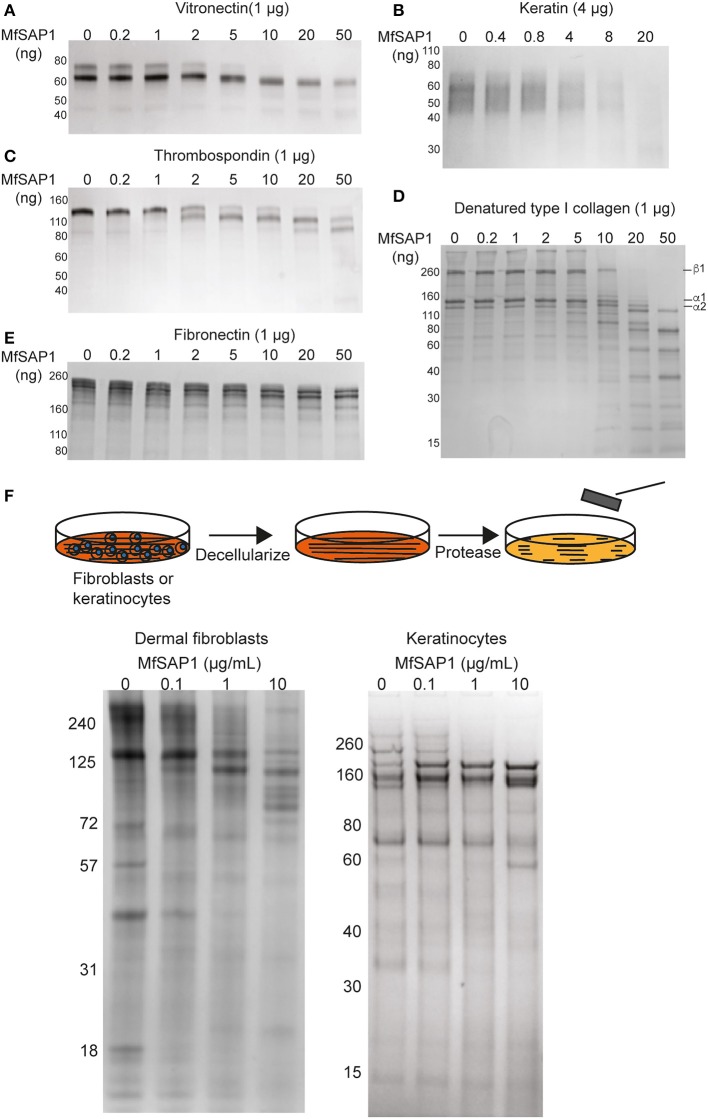
MfSAP1 degrades human extracellular matrix proteins. **(A–E)** ECM protein degradation, as assessed by SDS-PAGE, after MfSAP1 was incubated with each purified ECM protein at varying substrate to enzyme ratios. **(F)** Cartoon of the decellularization and protease treatment (top). Degradation of whole dermal fibroblast (left) and keratinocyte (right) ECM at different MfSAP1 concentrations by SDS-PAGE analysis. Representative SDS-PAGE images are shown for a total of at least 2 independent experiments.

*Malassezia* is the most abundant fungal family on the human skin at all sites except the feet (Belkaid and Segre, 2014). We therefore reasoned that the native substrates of MfSAP1 are likely extracellular proteins present on the human skin surface. To assess whether MfSAP1 processes common human extracellular matrix (ECM) proteins expressed by epidermal and dermal cells, we incubated MfSAP1 at varying enzyme to substrate ratios with purified ECM proteins at physiologically relevant condition of 34°C, pH 5 for 4 hrs. We observed strong degradation of vitronectin, human epidermal keratin, thrombospondin, and fibronectin at low enzyme to substrate ratios (Figures 3A–E). This is especially evident for keratin isolated from human epidermal culture, where we observed cleavage of keratin at 1,000-fold less MfSAP1. When we incubated MfSAP1 with type I collagen, the most abundant ECM protein in the dermal matrix, we observed only weak proteolysis of this matrix protein (Supplementary Figure 2). However, when the collagen was heat-denatured, MfSAP1 is able to degrade this ECM protein rapidly (Figure 3D). This indicates that MfSAP1 is unable to act on collagen I in its native, triple helix conformation. However, denatured collagen strands are accessible to MfSAP1 proteolysis. Degradation of collagen IV, a component of the epidermal-dermal junction (basement membrane) layer, proceeded similarly to that of type I collagen and denatured collagen IV was also cleaved more readily than native collagen IV (Supplementary Figure 2).

To assess MfSAP1's effect on intact native epidermal ECM and dermal ECM, we cultured N/TERT-1 keratinocytes and primary human dermal fibroblasts, decellularized these cultures, isolated the ECM produced *de novo*, and treated this ECM with MfSAP1 (Figure 3F). To increase fibroblast secretion of ECM we used macromolecular crowding to enhance collagen deposition (Lareu et al., 2007). We observed that ECM produced by both keratinocyte and fibroblast were rapidly degraded with only a short exposure to MfSAP1 at concentrations that are comparable to the concentrations of MfSAP1 secreted in culture. The sensitivity of *de novo* synthesized dermis/epidermis ECM was apparent even with low concentrations (0.1 μg/ml) of MfSAP1 (Figure 3F).

### Assessment of MfSAP1 in Epithelial Wound Healing

**Figure 4:**
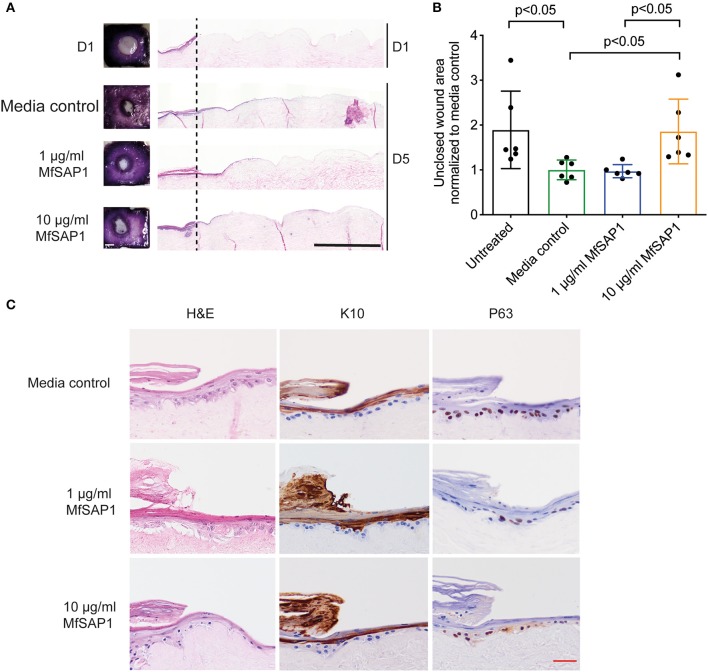
Effect of MfSAP1 on acute wound closure. **(A)** MTT staining (left) and hematoxylin and eosin (H&E, right) staining of the DED-HSE sections on Day 1 and Day 5. The dashed line indicates the edge of the punch biopsy. Black scale bar, 1 mm. **(B)** Quantification of the uncovered wound area as determined by MTT stain using ImageJ. Each wound area sample is normalized to the media control. Error bars represent standard deviation for *n* = 6 from a total of 3 experimental replicates with technical duplicates. **(C)** H&E and immunohistochemical analysis of the wound edge on Day 5 for each treatment condition. Representative images are shown from a total of 3 independent experiments. Red scale bar, 50 μm.

Our *in vitro* experiments demonstrated that MfSAP1 is able to efficiently cleave epidermal and dermal-associated ECM proteins. In order to determine whether this activity may have any effect on epidermal wound healing, we utilized an *in vitro* reconstructed 3-D skin model to test the effects of MfSAP1 on cutaneous wound healing. Primary human keratinocytes were cultured on de-epidermized dermis (DED) obtained from human donors creating a mature, full-thickness epidermis. A biopsy punch (4 mm) was used to completely remove the epidermis (Figure 4A, D1) leaving a reproducible acute wound on this DED-human skin equivalent (DED-HSE). MfSAP1 in control Full Green's media adjusted to pH 5 (for compatibility with MfSAP1's acidic pH optimal) was applied onto this wound daily for 4 days at two different protease concentrations (1 and 10 μg/ml) which we demonstrated above to be efficient in processing keratinocyte and fibroblast ECM proteins. Closure of the wound is quantitatively assessed using the conversion of MTT by the viable cells in the epithelial tongue (Figure 4A). We observed that treatment with 1 μg/ml of MfSAP1 had little effect on the epithelial migration compared to the control samples, as determined by wound area and lateral migration of the keratinocytes (Figure 4B, Supplementary Figure 3). We observed that acute wounds exposed to 10 μg/ml MfSAP1 were significantly slower to heal, as measured by uncovered wound area and lateral migration of keratinocytes (Figure 4B, Supplementary Figure 3). These data suggest that the proteolytic activity of MfSAP1 interferes with cell attachment to underlying ECM, thus inhibiting cell migration and epidermal wound closure.

We examined the histology of the keratinocytes in the wounded DED-HSE constructs using haematoxylin and eosin (H&E) (Figures 4A,C). Healing wound edges were characterized by an advancing wedge-shaped “epithelial tongue” in all treatment samples (Figure 4A). However, samples exposed to 10 μg/ml MfSAP1 exhibited reduced lateral cell migration and immature squamous differentiation compared to the media control treated samples, and to samples exposed to 1 μg/ml MfSAP1 (Figure 4C). To assess the effect on keratinocyte differentiation, we performed immunohistochemistry using the transcription factor p63 (un-differentiated cells), keratin 10 (suprabasal differentiated keratinocytes), and keratin 14 (basal un-differentiated keratinocytes) (Figure 4C, Supplementary Figure 3). We observed in the 10 μg/ml MfSAP1 treated DED-HSE constructs, most of the epithelial cells in the migrating tongue were undifferentiated basal keratinocytes. This contrasts with DED-HSE constructs exposed to the control media or 1 μg/ml MfSAP1, where layers of differentiated keratinocytes with high keratin 10 expression were evident adjacent to the wound margin (Figure 4C).

## Discussion

*Malassezia* is an integral component of the human skin microbiome as evident from its high prevalence across mammalian species (Gaitanis et al., 2012; Theelen et al., 2018). Recent studies have revealed *Malassezia* may also impact physiological events beyond the skin environment, in particular the human gastro-intestinal microbial ecosystem (Aykut et al., 2019; Limon et al., 2019). However, our appreciation and understanding of the molecular events involved in the interactions of *Malassezia* sp. with the host and other elements of the human microbiome is poor. In this study, we have focused on a secretory enzyme that has potential to act as a mediator in these interactions. We identified that the dominant protease secreted by *M. furfur* is MfSAP1, an aspartyl protease with high catalytic efficiency. MfSAP1 exhibits specific activity against a variety of elements of the dermal and epidermal ECM, a property that has important implications for cutaneous wound healing.

*Malassezia furfur* is among the earliest discovered of the *Malassezia* species. As such, many seminal works were performed using this species as an exemplar of *Malassezia* sp. in skin health and disease (Ashbee and Evans, 2002). As *M. furfur* is perhaps the most easily cultivated *Malassezia* species, it is frequently isolated from human skin in culture studies even though it is much less prevalent than *M. globosa* and *M. restricta* (Wu et al., 2015; Leong et al., 2019). Importantly, *M. furfur* CBS 14141 is one of only three strains of *Malassezia* that are currently amenable to genetic manipulation (Ianiri et al., 2016; Celis et al., 2017) and understanding the functions of secretory proteases in this strain opens up future opportunities for genetic approaches for further analysis.

When studying proteases secreted by *M. furfur* and *M. globosa*, we were surprised to recognize that *M. furfur* has a reduced number of secreted aspartyl proteases, but has an expanded number of secreted serine proteases. Nonetheless, secreted protease activity from *M. furfur* is dominated by a homolog of the previously characterized MgSAP1. The fact that the extracellular protease activity is dominated by just one secreted aspartyl protease in these two species suggests that homologs of MfSAP1 and MgSAP1 may also dominate the secreted protease activity repertoire of other *Malassezia* species. That homologous aspartyl protease has been retained in multiple species strongly implies that this protease family has integral functions across all members of the *Malassezia* family. Interestingly, while MfSAP1 has similarities with MgSAP1 in terms of protease expression and cleavage of the internally quenched substrates, MfSAP1 is catalytically more efficient and cleaves a wider range of substrates. By implication, at the same given concentration, MfSAP1 is able to process substrates at greater efficiency than MgSAP1. Furthermore, while MgSAP1 protease activity is tightly controlled at the post-translational level, and expression peaks during late log phase of planktonic growth, MfSAP1 is constitutively secreted during all stages of growth. Taken together, these observations highlight the importance of characterizing the activity of MfSAP1; despite its low abundance it is functionally relevant to the integrity and repair of human skin.

In healthy skin, the stratum corneum together with the tight junctions in the stratum granulosum forms a barrier against external agents (Matsui and Amagai, 2015). Given that *Malassezia* sp. are skin residing fungal species, we reasoned that the native substrates of MfSAP1 was likely to include molecular species present in the host's epidermal and dermal layers. As such, we considered that the relevance of MfSAP1's ECM processing activities should be considered only when the epidermal barrier is compromised, such as in an acute wound. We examined the ability of MfSAP1 to cleave a wide range of skin-associated ECM proteins *in vitro*, especially those associated with the basal keratinocytes. Key components of the epidermis and dermis ECM, including vitronectin, human epidermal keratin, thrombospondin and fibronectin are cleaved by MfSAP1 at low enzyme to substrate ratios. When present at high concentration, MfSAP1 was shown to attenuate re-epithelization, thus retarding wound healing. However, we recognize that wound healing is a complex process involving many factors contributed by the dermal layer, microvasculature, innate and adaptive immune systems; these are absent in our *in vitro* DED-HSE model. Nevertheless, in this model of partial-thickness acute wounding, we demonstrated that MfSAP1 may be part of this complex mix *in vivo*, and potentially attenuate wound healing through the degradation of key ECM components.

In conclusion, we have demonstrated in this study that *M. furfur*, a component of the human skin microbiome produces an aspartyl protease that has strong skin ECM degradation activity. Combined with our previous study of MgSAP1, it is evident that this aspartyl protease and its homologs likely modify the skin environment through degrading both human and bacteria associated substrates.

## Data Availability Statement

All datasets generated for this study are included in the article/Supplementary Material.

## Author Contributions

SP, DL, TD, and HL designed the experiments. HL, JG, and WC performed the protease isolation and characterization. SP, CF, SG, PL, and BS performed the DED-HSE experiments. SP, CF, and HL analyzed the data. CF and HL wrote the manuscript.

### Conflict of Interest

The authors declare that the research was conducted in the absence of any commercial or financial relationships that could be construed as a potential conflict of interest.
