# Supplementary material for: Identification of Malassezia furfur Secreted Aspartyl Protease 1 (MfSAP1) and Its Role in Extracellular Matrix Degradation
Source: Front Cell Infect Microbiol. 2020 Apr 9;10:148. doi: 10.3389/fcimb.2020.00148 (PMC7161080; doi:10.3389/fcimb.2020.00148)
Supplement: Supplementary file 1 [file Data_Sheet_1.docx]

Supplementary Material for Identification of *Malassezia furfur* secreted aspartyl protease 1 (MfSAP1) and its role in extracellular matrix degradation

# Supplementary Materials and Methods

**Peptide sequence of FRET-substrates**

The following list of substrates (with the CPC Scientific catalogue number) are used:

| **Substrate** | **Catalogue** | **Sequence** |
| --- | --- | --- |
| S1 | AMYD-109 | Mca-SEVNLDAEFRK(DNP)-RR-NH2 |
| S2 | AMYD-111 | Mca-RPPGFSAFK(DNP)-NH2 |
| S3 | AMYD112 | Mca-HQKLVFFAK(DNP)-NH2 |
| S4 | AMYD114 | Mca-EVKMDAEFK(DNP)-NH2 |
| S5 | AMYD103 | Mca-VNLDAEF-Lys(DNP)-NH_2_ |
| S6 | AMYD105 | Mca-SEVNLDAE-Dap(DNP)-NH_2_ |
| S7 | AMYD108 | Mca-SEVNLDAEF-K(DNP)-NH_2_ |
| S8 | AMYD110 | Mca-SEVKMDAEFR-K(DNP)-RR-NH_2_ |
| S9 | MMPS024 | Mca-RPKPYANvaWMK(DNP)-NH2 |
| S10 | MMPS009 | Mca-PLGL-Dap(DNP)-AR-NH_2_ |
| S11 | MMPS016 | Mca-P-Cha-G-Nva-HA-Dap(DNP)-NH_2_ |
| S12 | MMPS026 | Mca-RPKPVE-Nva-WRK(DNP)-NH_2_ |
| S13 | MMPS029 | Mca-KPLGL-Dap(DNP)-AR-NH_2_ |
| S14 | CAPS060 | Mca-VDQMDGWK-(DNP)-NH_2_ |
| S15 | CASP027 | Mca-YVADAP-Lys(DNP) |
| S16 | CASP028 | Mca-VDQVDGW-Lys(DNP)-NH_2_ |
| S17 | CASP059 | Mca-DEVDAP-Lys(DNP) |
| S18 | CASP068 | Mca-LEVDGWK(DNP)-NH_2_ |
| S19 | SUBS017 | Mca-GKPILFFRLK(DNP)-r-NH2 |

Mca: (7-methoxycoumarin-4-yl)acetyl, DNP: 2,4-dinitrophenol, Dap: 2,3-Diaminopropionic acid, Nva: Norvaline, r: D-arginine.

**Histology**

The DED-HSE samples were fixed in 10% neutral buffered formalin overnight at room temperature before embedding in parafﬁn. Sections of 7 μm thickness were cut and mounted onto glass slides. These sections were dewaxed in xylene and rehydrated in a series of decreasing concentrations of ethanol before hematoxylin and eosin (H&E) staining. 10X and 20X images were captured using the Nikon Eclipse Ts2R Inverted microscope and the Olympus BX43 microscope respectively.

**Immunohistochemistry**

The tissue sections were dewaxed and rehydrated as described above in the histology section. Samples were then immersed in citrate buffer pH6.0 (Electron Microscopy Sciences; 64142-08) and heat-induced epitope retrieval was performed using a 90⁰C water bath for 15 minutes. 1% hydrogen peroxide (Merck; 1.07209.0250) was used to quench endogenous peroxidase for 30 min. After quenching, the samples were blocked in 10% goat serum (Gibco; 16210072) at room temperature for 30 min before primary antibodies (see table below) were left on for 2 hours. Unbound primary antibodies were aspirated, tissue samples washed and secondary antibodies (DAKO; HRP-labelled Anti-mouse/rabbit polymer K4001/K4003) applied for 30min. Once the antibodies have been added, DAB substrate (DAKO; K3468) was used to visualize bound antibodies and the nuclei were counterstained with hematoxylin. The tissue sections were dehydrated through a series of increasing concentrations of ethanol to xylene to prepare for mounting. 20X images were captured using the Olympus BX43 microscope.

| **Antibody** | **Dilution Conditions** | **Company/CAT No** |
| --- | --- | --- |
| Keratin 10  Keratin 14  P63 | 1:200  1:25  1:50 | DAKO/DKO.M7002  (Supernatant)  Abcam/ab735 |

# Supplementary Figures

**Supplementary Figure 1.** Identification of MfSAP1. (A) Edman sequencing cycles of the two bands isolated from the pepstatin A-agarose affinity purification of *M. furfur* extracellular media. SDS-PAGE of the enriched protease with the two protein bands labeled as “top” or “bottom” is shown on the left and the corresponding Edman sequencing is shown on the right. (B) Protein sequence of MfSAP1, with the propeptide sequence shown in orange. The underlined peptide sequences are detected in the mass spectrometry of the in-gel trypsin digest of the enriched protease. (C) Activity of MfSAP1 at various pH, as determined by cleavage of substrate S12. (D) Michelis-Menten plot of MfSAP1 activity with substrate S12.

**Supplementary Figure 2.** MfSAP1 activity against human purified ECM proteins. (A) Rat tail type I collagen was incubated at various substrate to enzyme ratio (S/E) with MfSAP1 at two different pH and temperature for 4 h. (B) Human collagen IV and denatured collagen IV (C) were incubated with MfSAP1 at pH 5 at different substrate to enzyme ratio for 4 h at 34 ˚C.

**Supplementary Figure 3.** MfSAP1’s effect on re-epithelization. (A) Lateral migration of the epithelial surface as determined using ImageJ quantification of the MTT stains of the DED-HSE sections. The average of 4 measurements per section was taken and normalized to the media control. Error bars represent standard deviation for n=6 from a total of 3 experimental replicates with technical duplicates. (B) Immunohistochemical stain of keratin 14 for each treated DED-HSE section. Representative images are shown from a total of 3 experimental replicates.
